# Inflammation in Bipolar depression: triggers and targets

**DOI:** 10.1192/j.eurpsy.2025.162

**Published:** 2025-08-26

**Authors:** M. Leboyer

**Affiliations:** 1 Creteil, Paris Est University (UPEC); 2 Translational NeuroPsychiatry, INSERM; 3 Fondation FondaMental, Créteil, France

## Abstract

**Abstract:**

Repeated reports of association of low-grade inflammation in bipolar disorder led to exploration of the causes and consequences of this inflammatory background, now thought to be due to interaction between environmental factors such as infections, stress, pollution, unhealthy life style with immune-genetic background.

Association with functional gene variants of Toll-Like Receptor genes (TLR, NOD), possibly explain diminished response to infections observed in bipolar disorder (rev in Oliveira et al, 2017) association while mitochondrial genes (Angrand et al, BBI, 2021), HLA-E and HLA-G (Boukouaci et al, 2021) contribute to maintenance of inflammation. Association with particular HLA haplotypes very likely explains pro inflammation and auto-immune induction observed particulary in bipolar patients with suicidal behavior and/or with rapid cycling (Tamouza et al, 2020). While HLA haplotypes, in particular when they are associated to anti-inflammatory effect have also been found to be associated with good lithium response (Leclerc et al, 2021).

Systemic inflammation and persistent infections activate different pathways paving the way to biomarker-guided personalized medicine. For example the identification of **“**autoimmune psychosis**”** defined by presence of anti-neuronal antibodies in psychosis and in bipolar disorder (Jezequel et al, 2017). Systemic inflammation induced by microbial infection and/or psychosocial factors can also be at the origin of the activation of human endogenous retrovirus (HERV-W) in patients with bipolar disorder (Tamouza et al, 2021). One last example can be found in the immune-metabolic abnormalities that pave the way to metabolic syndrome associated with psychiatric disorders. Various mitochondrial dysfunctions have recently been reported including deregulated bioenergetics and mitochondrial DNA alterations in bipolar disorder, underlying depressive and manic episodes and their association with metabolic syndrome.

Findings accumulated so far favour the consideration of cellular and molecular targets for the treatment of specific subgroups of bipolar disorders. This is the case for clinical trials of the efficacy of anti-inflammatory treatment in patients with blood auto-antibodies against brain receptors. Or for low dose IL-2 therapy in bipolar depression based on the hypothesis that IL-2 is a differentiation factor for T cells and low dose IL-2 combats premature T cell aging and induce the production of new naïve T cells from the thymus particularly T regulator cells (Leboyer et al, 2025). Another example comes from drugs that boost mitochondrial function in bipolar depression (Khandra et al, 2023).

**Disclosure of Interest:**

None Declared

